# Highly Pathogenic Porcine Reproductive and Respiratory Syndrome Virus Nsp4 Cleaves VISA to Impair Antiviral Responses Mediated by RIG-I-like Receptors

**DOI:** 10.1038/srep28497

**Published:** 2016-06-22

**Authors:** Chen Huang, Yinping Du, Zhibin Yu, Qiong Zhang, Yihao Liu, Jun Tang, Jishu Shi, Wen-hai Feng

**Affiliations:** 1 State Key Laboratory of Agrobiotechnology, Beijing 100193, China; 2 Ministry of Agriculture Key Laboratory of Soil Microbiology, Beijing 100193, China; 3 Department of Microbiology and Immunology, College of Biological Sciences, China Agricultural University, Beijing 100193, China; 4 Department of Basic Veterinary Medicine, College of Veterinary Medicine, China Agricultural University, Beijing 100193, China.; 5 Department of Anatomy and Physiology, College of Veterinary Medicine, Kansas State University, Manhattan, KS, United States.

## Abstract

Porcine reproductive and respiratory syndrome virus (PRRSV) is one of the most significant etiological agents in the swine industry worldwide. It has been reported that PRRSV infection can modulate host immune responses, and innate immune evasion is thought to play a vital role in PRRSV pathogenesis. In this study, we demonstrated that highly pathogenic PRRSV (HP-PRRSV) infection specifically down-regulated virus-induced signaling adaptor (VISA), a unique adaptor molecule that is essential for retinoic acid induced gene-I (RIG-I) and melanoma differentiation associated gene 5 (MDA5) signal transduction. Moreover, we verified that nsp4 inhibited IRF3 activation induced by signaling molecules, including RIG-I, MDA5, VISA, and TBK1, but not IRF3. Subsequently, we demonstrated that HP-PRRSV nsp4 down-regulated VISA and suppressed type I IFN induction. Importantly, VISA was cleaved by nsp4 and released from mitochondrial membrane, which interrupted the downstream signaling of VISA. However, catalytically inactive mutant of nsp4 abolished its ability to cleave VISA. Interestingly, nsp4 of typical PRRSV strain CH-1a had no effect on VISA. Taken together, these findings reveal a strategy evolved by HP-PRRSV to counteract anti-viral innate immune signaling, which complements the known PRRSV-mediated immune-evasion mechanisms.

Type I interferons (IFN-I) play vital roles in innate immune responses against invading viruses. The initiation of IFN-I production relies on host pathogen recognition receptors (PRRs) that recognize pathogen-associated molecular patterns (PAMPs)^1^. Membrane-bound Toll-like receptors (TLR), especially TLR3, TLR7/8 and TLR9, detect viral products on the cell surface or in endosomes^2^. Cytoplasmic RIG-I-like receptors (RLRs), including retinoic acid-induced gene I (RIG-I), melanoma differentiation associated gene 5 (MDA5) and laboratory of genetics and physiology 2 (LGP2), recognize viral or other xenogeneic nucleic acid in the cytoplasm^3^,^4^. RIG-I and MDA5 consist of caspase activation and recruitment domains (CARD), an ATPase containing DEAD box helicase (DEAD helicase) and a C-terminal domain (CTD)^5^,^6^. Activation of RIG-I or MDA5 interacts via CARD-CARD interactions with virus-induced signaling adaptor (VISA) protein (also known as MAVS, IPS-1, or Cardif), the essential signaling adapter protein of the RLRs^7^,^8^,^9^,^10^. These interactions serve to relocate the RLRs to mitochondria membranes and initiate the formation of VISA signalosome with the downstream signaling molecules TANK-binding kinase 1 (TBK1) and IκB kinase-ε (IKKε), which phosphorylate and activate IRF3 and IRF7^11^. VISA also recruits the Fas-associated death domain protein (FADD), which activate caspase-10 and caspase-8, driving NF-κB activation^12^. Upon activation, IRF3/7 and NF-κB translocate to the nucleus to trigger transcription of a variety of cytokines, including IFN-I. Following production and secretion, IFN-I bind to the type I IFN receptor (IFNAR) and initiate the Janus kinase signal transducer and activator of transcription (JAK-STAT) pathway, resulting in the expression of hundreds of IFN stimulated genes (ISGs)^13^,^14^. IFN-I responses lead to a remarkable antiviral state that resists virus infection.

Porcine reproductive and respiratory syndrome virus (PRRSV) is an enveloped positive-stranded RNA virus, which belongs to *Arteriviridae* family^15^. Since identified in 1991, PRRSV has been one of the most important infectious agents in swine industry worldwide^16^. In 2006, a highly pathogenic PRRSV (HP-PRRSV) strain causing porcine high fever syndrome (PHFS) was reported in China, leading to significant economic losses^17^,^18^. PRRSV genome is approximately 15,000 nucleotides in length with at least 10 open reading frames that encode two polyprotein precursors (pp1a and pp1ab) and 8 structural proteins^19^. Upon infection, pp1a and pp1ab are processed into at least 16 nonstructural proteins: nsp1α, nsp1β, nsp2-6, nsp2TF, nsp2N, nsp7α, nsp7β and nsp8-12^20^,^21^,^22^,^23^. In addition to their activities in viral replication processing, some of these proteins participate in modulating host immune responses.

Several studies suggest that RIG-I and MDA-5 play a pivotal role in countering infection by arteriviruses^24^,^25^. In addition, it has been reported that poly(I:C) and IFNβ limit arterivirus infection^26^,^27^. Therefore, it is not surprising that arteriviruses have evolved mechanisms to escape antiviral innate immune responses. For example, several arteriviruses and their proteins cleave or interact with signaling molecules of IFN-I responses^28^,^29^. Recently, we demonstrated that HP-PRRSV and its protein nsp4 antagonized type I IFN expression by cleaving NF-κB essential modulator (NEMO) to block NF-κB signaling pathways^30^. Although this involves the nsp4 protein that down-regulates NF-κB, the precise role of PRRSV nsp4 on evading IFN-I responses remains to be further defined.

In the present study, we showed that IFNβ induction and ISGs expression were inhibited by HP-PRRSV through negatively regulating IRF3 signaling pathway in infected cells. Subsequently, we found that HP-PRRSV reduced the expression of VISA, an essential adaptor in RLR signaling pathway. Such effect required the nsp4 protein, which mediates VISA cleavage and releases it from mitochondrial membrane. However, a typical PRRSV strain CH-1a did not down-regulate VISA expression, and CH-1a nsp4 also had no effect on VISA. This discovery suggests that nsp4 contributes to HP-PRRSV pathogenesis through the inhibition of VISA-dependent signaling pathway.

## Results

### IFNβ induction and signaling is inhibited by HP-PRRSV infection

**Figure 1:**
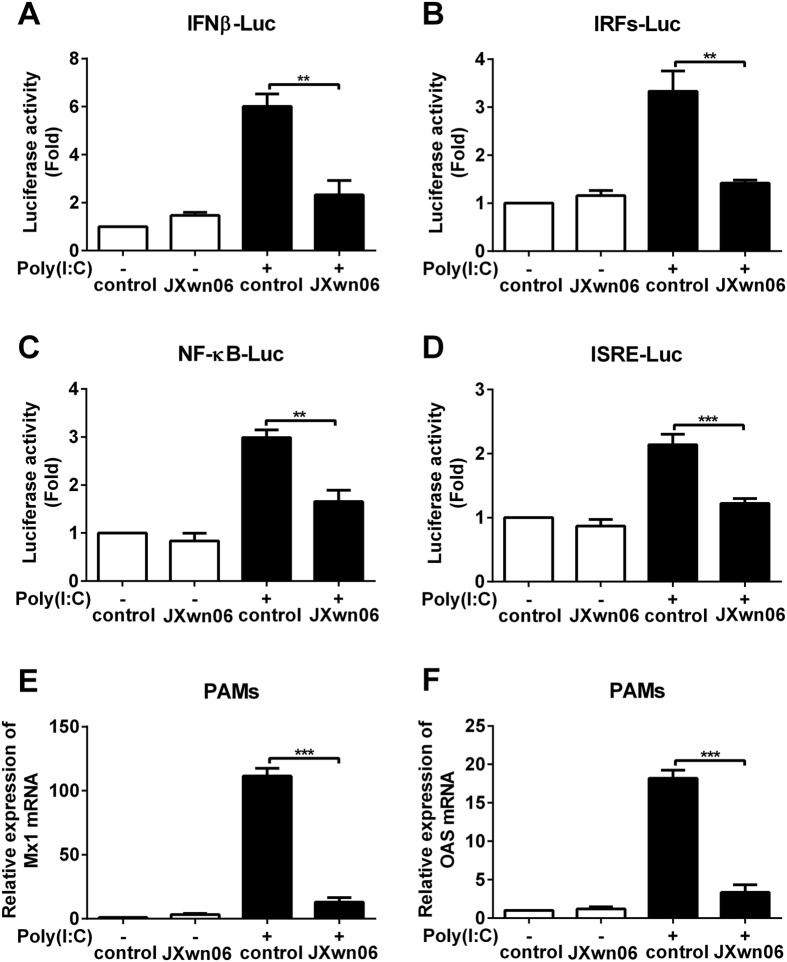
HP-PRRSV infection inhibits IFNβ induction and signaling. (**A–D**) Marc-145 cells were transfected with pGL3-IFNβ-Luc (**A**), pGL3-IRFs-Luc (**B**), pGL3-NF-κB-Luc (**C**) or pGL3-ISRE-Luc (**D**), and pRL-TK. pRL-TK was used as an internal control of transfection efficiency. Six hours later, cells were mock infected or infected with HP-PRRSV (JXwn06) at a multiplicity of infection (MOI) of 0.1 for 24 h, and then transfected with or without poly(I:C) for 8 h. Total cell lysates were assayed for luciferase activities. (**E,F**) Porcine alveolar macrophages (PAMs) were mock infected or infected with HP-PRRSV at an MOI of 0.1 for 24 h, and then treated with or without poly(I:C) (10 μg/ml) for 6 h. Total RNAs were extracted and qPCR was performed for analyzing the expression of Mx1 (**E**) and OAS (**F**). GAPDH was used as an internal control. Data were representative of three independent experiments (mean ± SD). Statistical analysis was performed by Student’s *t* test. **P < 0.01; ***P < 0.001.

In the previous study, we verified that HP-PRRSV inhibited the production of IFNα and IFNβ in PAMs by qPCR^30^. To identify the transcription factors which participate in the effect of HP-PRRSV on IFNβ induction and signaling, Marc-145 cells were transfected with IFNβ, IRFs, NF-κB or ISRE response elements luciferase reporter plasmid. Six hours later, Marc-145 cells were infected with HP-PRRSV strain JXwn06, and then transfected with or without poly(I:C). At 8 h post-transfection, luciferase activities were examined. As expected, HP-PRRSV infection reduced IFNβ promoter activity induced by poly(I:C) (Fig. 1A). In addition, IRFs, NF-κB and ISRE responsive promoter activities induced by poly(I:C) were also affected by HP-PRRSV infection with the down-regulation of 57%, 45% and 43% compared to that of mock infection, respectively (Fig. 1B–D). We also infected porcine alveolar macrophages (PAMs) with HP-PRRSV, and analyzed the mRNA levels of ISGs using qPCR. As shown in Fig. 1E and F, HP-PRRSV significantly suppressed the expression of myxovirus resistance 1 and 2′,5′-oligoadenylate synthetase to 12% (Mx1) (Fig. 1E) and 18% (OAS) (Fig. 1F) of that induced by poly(I:C). These data suggest that HP-PRRSV infection antagonizes IFNβ induction and downstream signaling pathway.

### HP-PRRSV nsp4 blocks IRF3 signaling pathway

**Figure 2:**
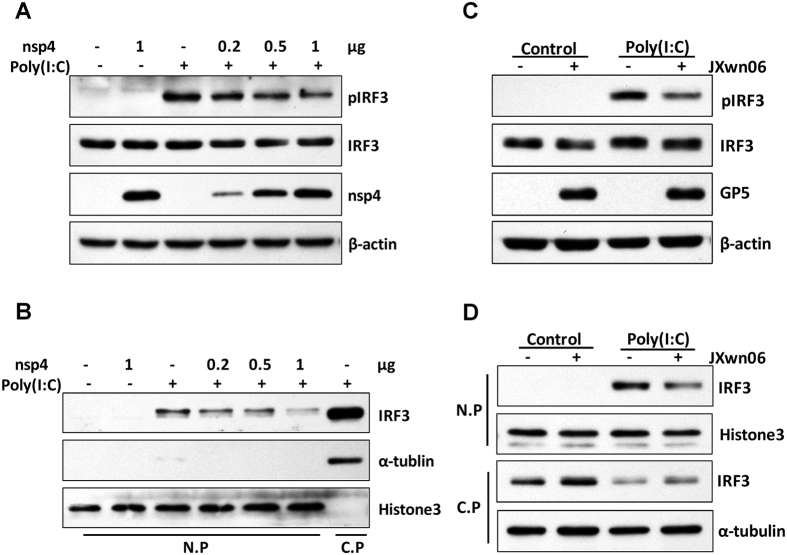
HP-PRRSV nsp4 antagonizes IRF3 signaling pathway. (**A,B**) Control vector or nsp4 expression plasmid was transfected into 3D4/21 cells. Twenty-four hours later, cells were transfected with or without poly(I:C) for 2 h. (**C,D**) PAMs were mock infected or infected with HP-PRRSV (JXwn06) at an MOI of 0.1. Twenty-four hours post-infection, cells were treated with or without poly(I:C) (10 μg/ml) for 2 h. (**A,C**) Lysates of cells were subjected to Western blot analysis with antibodies against IRF3, pIRF3, nsp4,GP5 and β-actin, respectively. (**B,D**) Total cells were lysed and then nuclei protein (N.P) and cytoplasma protein (C.P) fractions were separated for detecting the distribution of IRF3. Histone3 and α-tubulin were set up as nuclei and cytoplasma control, respectively. Data were representative of three independent experiments.

IRF3 is the most significant transcription factor in IFNβ induction. To confirm and clarify whether and how nsp4 inhibits IRF3 signaling pathway, we analyzed several key steps of the IRF3 activation process, such as IRF3 phosphorylation and nuclear translocation, upon poly(I:C) treatment. As shown in Fig. 2A, treatment with poly(I:C) activated IRF3 phosphorylation in 3D4/21 cells. However, addition of nsp4 reduced the phosphorylation level of IRF3 in a dose-dependent manner, suggesting that nsp4 inhibits IFNβ induction upstream of IRF3 activation. To confirm this result, nuclei protein (N.P) and cytoplasma protein (C.P) fractions were separated for analyzing the distribution of IRF3 (Fig. 2B). The results showed that nsp4 expression led to a pronounced, dose-dependent decrease in poly(I:C)-induced IRF3 nuclear translocation.

Subsequently, we examined IRF3 phosphorylation during the course of HP-PRRSV infection. As expected, poly(I:C) activated IRF3 phosphorylation in mock infected cells. However, the activation was remarkably impaired in cells infected with HP-PRRSV (Fig. 2C). In consistent with this result, nuclear translocation of IRF3 was also affected. As shown in Fig. 2D, the level of IRF3 presented in the nuclear fraction increased in the presence of poly(I:C), whereas nuclear translocation of IRF3 was down-regulated in PRRSV-infected PAMs. Taken together, these data show that HP-PRRSV and its protein nsp4 interfere with poly(I:C) activated IRF3 signaling pathway by reducing IRF3 phosphorylation and translocation to the nucleus.

### HP-PRRSV nsp4 disrupts RIG-I like receptor signaling pathway

**Figure 3:**
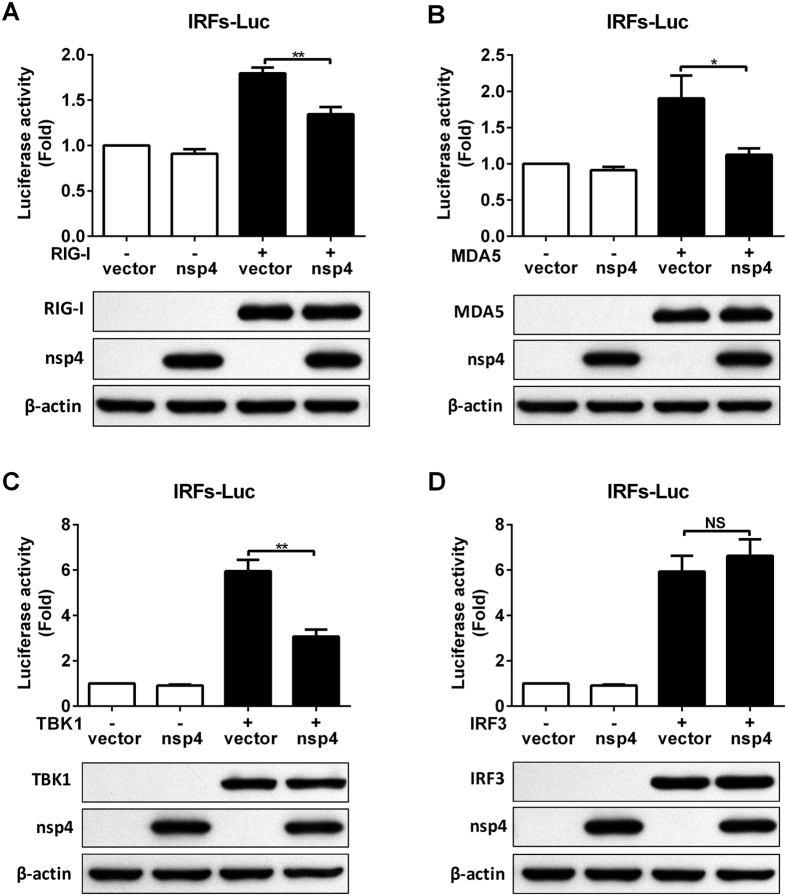
Effect of nsp4 on RIG-I like receptor signaling pathway. (**A–D**) HeLa cells were co-transfected with nsp4 and pGL3-IRFs-Luc along with RLR signaling molecule expression vector, RIG-I (**A**), MDA5 (**B**), TBK1 (**C**), or IRF3 (**D**). A plasmid expressing pRL-TK was used as a control. Twenty-four hours later, cell lysates were assayed for luciferase activities. In addition, lysates of cells were subjected to Western blot analysis with antibodies against Flag, c-Myc and β-actin to show the expression of these signaling molecules and nsp4. Data were representative of three independent experiments with triplicate samples (mean ± SD). Statistical analysis was performed by Student’s *t* test. *P < 0.1; **P < 0.01; NS, not significant.

RIG-I and RLR protein family are key cytoplasmic receptors that are implicated in pathogen sensing of virus infection to initiate and modulate antiviral immune responses^3^,^4^. Arteriviruses were shown to be primarily detected by MDA5, while RIG-I might also be involved under some circumstances^25^. Given the pivotal role of RLRs in mediating IFN-I production and ISGs expression, we further investigated whether over-expression of HP-PRRSV nsp4 inhibits RLRs-mediated signaling. Over-expression of RIG-I or MDA5 stimulated IRFs responsive promoter activity compared with that of the vector control (Fig. 3A,B). However, activation of IRFs responsive promoter by RIG-I or MDA5 was significantly down-regulated to 75% and 60% in the presence of nsp4, respectively. Similar results were obtained when IRFs responsive promoter was activated by overexpression of TBK1, the downstream kinase for RIG-I/MDA5 (Fig. 3C). IRFs responsive promoter luciferase activity induced by TBK1 was suppressed about 48% by nsp4. In contrast, activation of IRFs responsive promoter by over-expression IRF3 was not affected by nsp4 (Fig. 3D). These results suggest that HP-PRRSV nsp4 disrupts RLR signaling pathway at a step upstream of IRF3.

### HP-PRRSV nsp4 inhibits IFN responses induced by VISA

**Figure 4:**
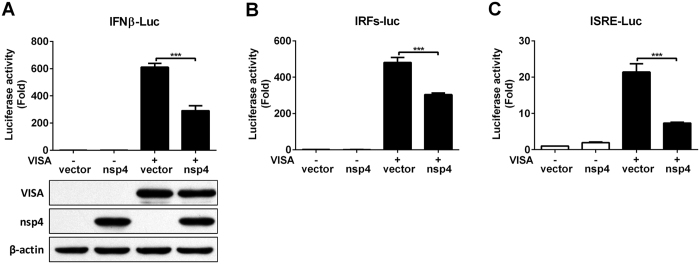
HP-PRRSV nsp4 suppresses IFN responses induced by VISA. (**A–C**) nsp4 and VISA expression plasmids were transfected into 3D4/21 cells together with pGL3-IFNβ-Luc (**A**), pGL3-IRFs-Luc (**B**) or pGL3-ISRE-Luc (**C**), and pRL-TK. pRL-TK was used as an internal control of transfection efficiency. At 24 h after transfection, cells were harvested for luciferase activity detection. Lysates of cells from panel A were subjected to Western blot analysis with anti-Flag, c-Myc and β-actin antibodies to detect VISA and nsp4 expression. Data are mean ± SD from three independent experiments with triplicate samples. Statistical analysis was performed by Student’s *t* test. ***P < 0.001.

Sensing of virus by the RLRs engages a complex signaling cascade that utilizes VISA adapter protein to initiate the innate host antiviral and inflammatory responses against pathogen infection^31^. VISA is central to RLR signaling pathway and orchestrates the ordered recruitment of various signaling molecules to create an antiviral platform^32^,^33^. To determine whether nsp4 inhibits IFN responses induced by VISA, we transfected expression vectors encoding nsp4 and VISA into 3D4/21 cells together with a luciferase reporter driven by the IFNβ promoter, IRFs responsive promoter or ISRE responsive promoter. As shown in Fig. 4A, over-expression of VISA significantly up-regulated IFNβ promoter activity to about 600-fold compared with that of the vector control. However, co-transfection of nsp4 could result in a 52% decrease of luciferase activity relative to transfected with VISA only. In consistent with this observation, IRF3 and ISRE responsive promoter activity was stimulated in cells transfected with VISA construct, while the luciferase activity was suppressed about 37% and 66% in the presence of nsp4, respectively (Fig. 4B,C). These results suggest that HP-PRRSV nsp4 blocks IFNβ induction and downstream signaling by VISA.

### HP-PRRSV nsp4 reduces endogenous VISA expression

**Figure 5:**
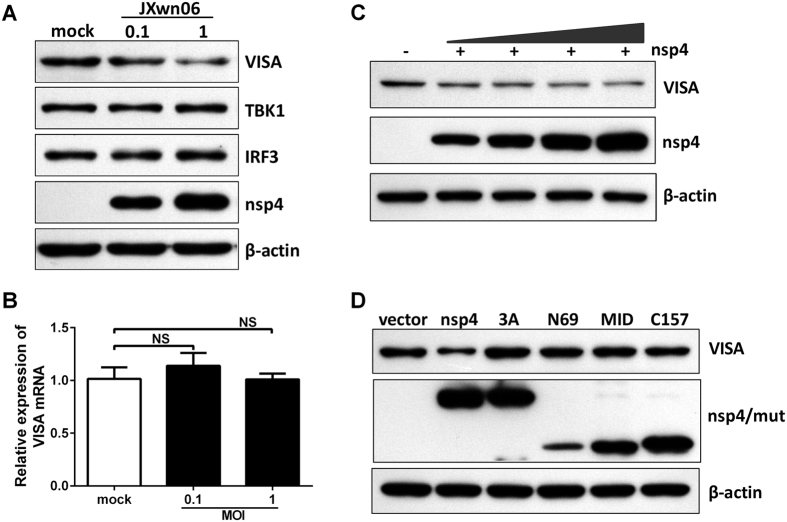
HP-PRRSV nsp4 reduces endogenous VISA expression. (**A,B**) PAMs were mock infected or infected with HP-PRRSV (JXwn06) at an MOI of 0.1 or 1. Thirty-six hours later, total cell lysates were analyzed by Western blot to examine the levels of VISA, TBK1, IRF3, nsp4 and β-actin (**A**). Total RNAs were extracted and qPCR was performed for analyzing the RNA level of VISA (**B**). GAPDH was used as an internal control. (**C,D**) 3D4/21 cells were transfected with increasing amounts of nsp4 expression plasmids (**C**) or nsp4 mutant expression plasmids (**D**). At 24 h after transfection, cells were harvested and performed the expression of VISA and nsp4 by Western blot analysis. β-actin was set up as a loading control. Data were representative of three independent experiments (mean ± SD). Statistical analysis was performed by Student’s *t* test. NS, not significant.

The data presented above show that HP-PRRSV nsp4 might inhibit RLR signaling at a step upstream of IRF3. Considering that VISA plays a pivotal role in RLR signaling and a variety of viruses evolve mechanisms to disrupt VISA, we then investigated whether HP-PRRSV infection affected VISA expression. PAMs were mock infected or infected with HP-PRRSV strain JXwn06. Thirty-six hours post-infection, cells were harvested and examined by Western blot analysis. As shown in Fig. 5A, the level of endogenous VISA protein decreased in a dose-dependent manner. In contrast, TBK1 and IRF3 exhibited no reduction in HP-PRRSV-infected PAMs. To examine at which level HP-PRRSV affects VISA expression, the mRNA level of VISA was analyzed using qPCR. Result showed that HP-PRRSV had no effect on the mRNA level of VISA (Fig. 5B), implicating that HP-PRRSV infection might affect the protein level of VISA.

Next, we analyzed the effect of HP-PRRSV nsp4 on VISA expression. Expression vector encoding nsp4 was transfected into porcine 3D4/21 cells and Western blot analysis was performed. As shown in Fig. 5C, nsp4 reduced the level of endogenous VISA in a dose-dependent manner when over-expressed in cells. This coincided well with the progress of HP-PRRSV infection. To determine whether the protease activity of nsp4 is required for this reduction, we constructed a series of nsp4 mutants, including mutation of the canonical catalytic triad of His39–Asp64–Ser118 (3A) and one of three domains located at amino acids 1–69 (N69), 89–153(MID), and 157–199 (C157), respectively^34^. Results showed that these protease-dead mutants completely lost its ability to down-regulate the protein level of VISA (Fig. 5D). Taken together, these results indicate that HP-PRRSV, as well as its protein nsp4, reduces endogenous VISA level.

### VISA is cleaved from mitochondria by HP-PRRSV nsp4

**Figure 6:**
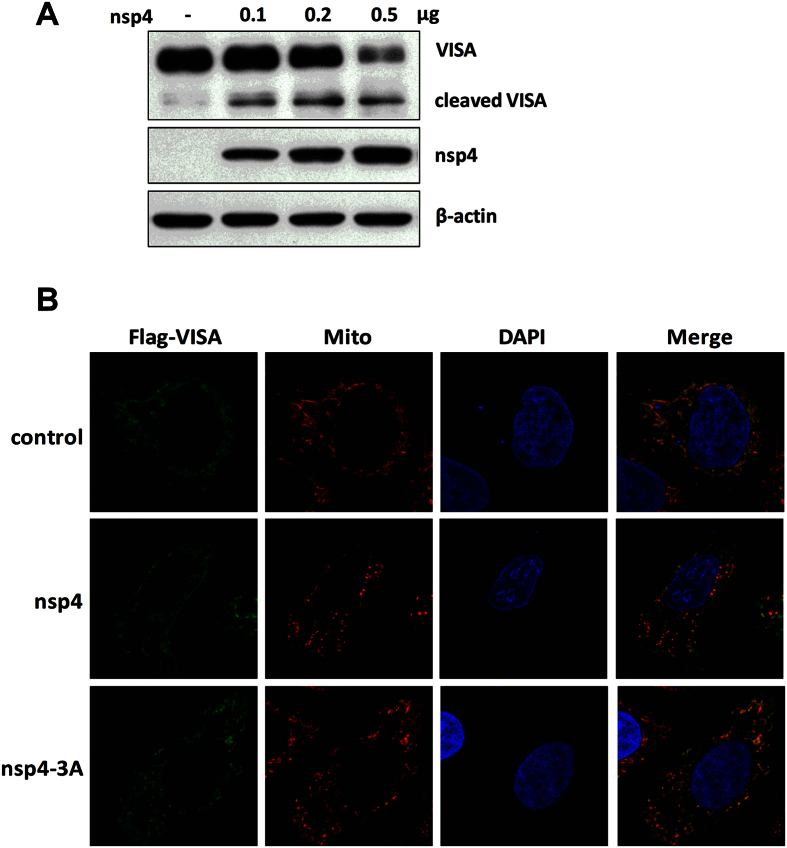
HP-PRRSV nsp4 mediates VISA cleavage from mitochondrial membrane. (**A–C**) N-Flag-tagged porcine VISA expression plasmid was co-transfected into HeLa cells with empty vector, increasing amounts of nsp4 expression plasmids or nsp4-3A expression plasmid for 24 h. (**A**) Total cells were collected and Western blot analysis for VISA and nsp4 expression was performed by incubating with specific antibodies against Flag or c-Myc, respectively. β-actin was set up as a loading control. (**B**) Cells were then fixed, stained for VISA and mitochondria using the indicated antibodies, and the localization of these proteins was visualized by confocal microscopy (VISA: green; mitochondria: red; nucleus: blue). All images represent 3 independent experiments.

The above findings that the protease activity is critical for HP-PRRSV nsp4-mediated reduction of endogenous VISA raise the possibility that nsp4 might target VISA for cleavage. To verify this possibility, we cloned porcine VISA gene into a mammalian expression vector with N-Flag tag. The expression vector encoding VISA was co-transfected into HeLa cells with empty or HP-PRRSV nsp4-expressed vector. As shown in Fig. 6A, the protein abundance of full-length Flag-VISA was decreased as the level of HP-PRRSV nsp4 increased. This was accompanied by the appearance of a smaller anti-Flag-reactive band, presumably a VISA cleavage product.

VISA is primarily localized on the outer mitochondrial membrane, and this sub-cellular localization is essential for its function in RLR antiviral signaling^35^. We therefore investigated whether any changes to the cellular distribution of VISA occurred during nsp4 expression. Expression vector encoding Flag-VISA along with c-Myc-nsp4 or nsp4-3A was transfected into HeLa cells. Twenty-four hours post-transfection, cells were stained with the corresponding antibodies followed by imaging with a laser scanning confocal microscope. The results showed that VISA co-localized with a mitochondrial marker in control cells. When wild-type nsp4 was co-expressed with VISA, the majority of VISA became cytosolic, as revealed by Flag-antibody that detects the N terminus of VISA. In sharp contrast, when nsp4 mutant (3A) and VISA were co-expressed, VISA co-localized on the mitochondrial membrane and revealed an extensive overlapping staining pattern (Fig. 6B). These data indicate that HP-PRRSV nsp4 mediates VISA cleavage from mitochondrial membrane.

### nsp4 of typical PRRSV strain CH-1a has no effect on VISA

**Figure 7:**
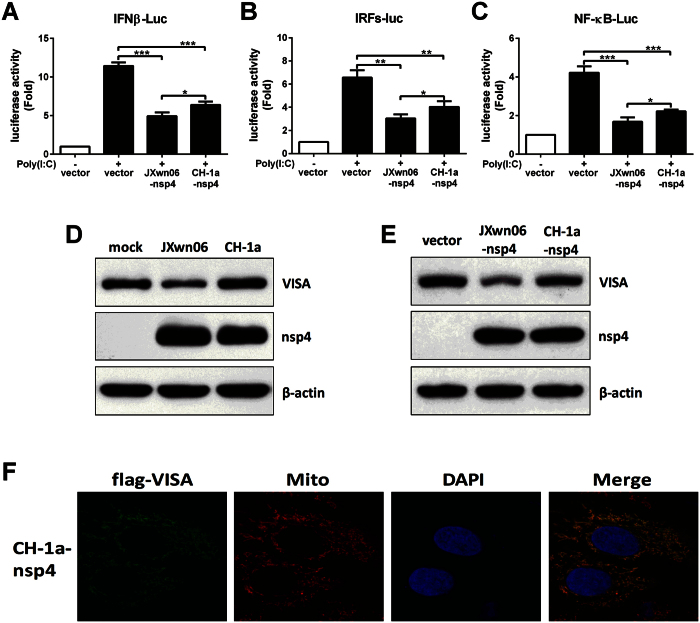
CH-1a nsp4 has no effect on VISA cleavage. (**A–C**) nsp4 of JXwn06 or CH-1a isolate expression plasmid was transfected into 3D4/21 cells along with pGL3-IFNβ-Luc (**A**), pGL3-IRFs-Luc (**B**) or pGL3-NF-κB-Luc (**C**), and pRL-TK. pRL-TK was used as an internal control of transfection efficiency. Twenty-four hours later, cells were stimulated with or without poly(I:C) (10 μg/ml) for 8 h and analyzed using dual-luciferase assay. (**D**) PAMs were mock infected or infected with JXwn06 or CH-1a at an MOI of 1 for 24 h, and cells were harvested and performed the expression of VISA and nsp4 by Western blot analysis. (**E**) 3D4/21 cells were transfected with JXwn06 nsp4 or CH-1a nsp4 expression plasmid for 24 h, and then cell lysates were analyzed by Western blotting with antibodies for VISA, nsp4 and β-actin. β-actin was set up as a loading control. (**F**) HeLa cell treatment and confocal microscopy were performed as described in the legend to Fig. 7B. Data are mean ± SD from three independent experiments. Differences were evaluated by Student’s *t* test. *P < 0.05; **P < 0.01; ***P < 0.001.

Recent study reported that nsp4 of different pathogenic PRRSV isolates exhibited differential inhibitory effect on IFNβ transcription activation^36^. To confirm these results, vectors encoding JXwn06 nsp4 or CH-1a nsp4 were transfected into 3D4/21 cells along with a luciferase reporter driven by the IFNβ promoter, IRFs responsive promoter or NF-κB responsive promoter. Compared to nsp4 of typical PRRSV strain CH-1a, nsp4 of HP-PRRSV strain JXwn06 had a greater ability to suppress IFNβ, IRFs and NF-κB luciferase activities induced by poly(I:C) (Fig. 7A–C). We then tried to verify whether the two PRRSV isolates have different effects on VISA. As shown in Fig. 7D, JXwn06 infection significantly down-regulated VISA expression, while CH-1a had no effect on VISA expression. In consistent with this observation, over-expression JXwn06 nsp4 reduced VISA expression, whereas over-expression of CH-1a nsp4 had no impact on the level of endogenous VISA protein (Fig. 7E). In addition, confocal microscopy analysis revealed that VISA co-localized on the mitochondrial membrane in the presence of CH-1a nsp4, indicating that CH-1a nsp4 has no ability to mediate the cleavage of exogenous porcine VISA (Fig. 7F). Taken together, these data show that nsp4 of different pathogenic PRRSV strains display various effects on VISA.

Collectively, these data demonstrate that HP-PRRSV nsp4 mediates the cleavage of VISA and dislodges it from the mitochondria, thus impairing IFNβ induction and RLR signaling. However, CH-1a nsp4 has no effect on VISA.

## Discussion

HP-PRRSV causes porcine high fever syndrome (PHFS), which is characterized by high transmission efficiency, high morbidity and high mortality. Modulated and hijacked host immune responses are thought to facilitate viral pathogenesis. Sensing cytoplasmic RNA to initiate the induction of IFN-I by RLRs is a vital part of the innate antiviral responses. Therefore, the RLR signaling pathway is targeted by numerous viruses. In this work, we show that HP-PRRSV, as well as its protein nsp4, disrupts IFN-I induction and downstream signaling by mediating the cleavage of VISA, thus blocking RLR antiviral signaling pathway. However, nsp4 of conventional PRRSV strain CH-1a has no effect on VISA.

IFN-I are a family of cytokines that represent one of the first lines of defense to limit viral propagation and spread^13^. Secreted IFN-I trigger the activation of JAK-STAT signaling and then induce a large array of ISGs, leading to a remarkable antiviral state in cells^37^. Many ISGs control viral infection by directly interfering with different stages of viral life cycles or modulating antiviral immune responses. For instance, Mx1 traps nucleocapsids and prevents endocytosis of incoming virus particles. OAS catalyzes the synthesis of 2′, 5′-oligoadenylates, resulting in the activation of RNaseL, which mediates host and viral RNA degradation. Degraded RNA can activate cytoplasmic PRRs, such as RIG-I and MDA5, to reinforce the innate antiviral immunity^38^,^39^. In addition, IFN-I have been shown to play a significant role in shaping the adaptive immunity by promoting the development of antigen-specific T and B lymphocyte responses and immunological memory^40^. In consideration of the formidable antiviral function of IFN-I, it is not surprising that many viruses have evolved mechanisms to either prevent IFN-I induction or block the downstream signaling. Previous studies have shown that PRRSV has evolved a variety of strategies to inhibit and manipulate IFN-I responses^41^,^42^. Kim *et al*. have reported that nsp1 mediates CREB-binding protein (CBP) degradation to inhibit IRF3 association with CBP in the nucleus, resulting in the inhibition of IFN-I transcriptional activation^43^. PRRSV nsp2 has the ability to inhibit NF-κB activation by suppressing IκBα degradation^44^. nsp2 also has the effect on interfering with ISG15 conjugation to cellular proteins, impairing function of the antiviral ISG^45^. In the present study, we further verified that HP-PRRSV antagonized the production of IFN-I by inhibition of IRF3 activation. HP-PRRSV infection reduced the protein level of VISA, resulting in the disruption of RLR downstream signal transduction. In addition, we showed that HP-PRRSV infection inhibited the expression of ISGs induced by poly(I:C) (Fig. 1E,F). Except the inhibition of IFN-I expression, another possible strategy used by HP-PRRSV could be to suppress IFN-I downstream signaling. To verify this hypothesis, we used IFNα to induce JAK-STAT signaling pathway, and found that ISGs transcription was suppressed by HP-PRRSV (data not shown). This finding is in consistent with a previous study, in which the typical PRRSV strain VR2385 is used^46^.

RLRs are essential cytoplasmic pathogen recognition receptors that impart recognition of RNA viruses across genera and virus families, including *Paramyxoviridae*, *Picornaviridae*, *Coronaviridae*, *Arteriviridae*, etc^24^. Anti-virus response by the RLRs relies on the VISA adaptor protein to engage a high-energy signalosome that initiates downstream activation of transcriptional responses, inducing expression of IFN-I and immune modulatory genes to control virus propagation and spread^32^. VISA plays a central role in the induction of antiviral and inflammatory immune responses. Additionally, VISA appears to be involved in the coordination of metabolic and apoptotic functions^47^. Previous study reveals that mice deficient in VISA fail to mount a robust IFN-I responses and are highly susceptible to viral infection^48^. Thus, targeting VISA is likely to be an evolutionarily conserved and effective mechanism to suppress IFN-I transcription by viruses. Hepatitis C virus (HCV) NS3-4A protease is shown to inhibit VISA by cleaving it from the mitochondrial membrane and preventing the induction of IFNβ^49^. Wei *et al*. demonstrates that Hepatitis B virus (HBV) X protein promotes polyubiquitin conjugation to VISA, resulting in its degradation^50^. Human Metapneumovirus M2-2 protein is shown to inhibit VISA signaling and IFNβ production by interacting with VISA, which prevents the recruitment of VISA to RIG-I^51^. Here, we demonstrated that HP-PRRSV nsp4 protease inhibited IFN-I induction and signaling induced by VISA over-expression (Fig. 4). Furthermore, we showed that nsp4 had the ability to inactivate VISA by cleaving and dislocating it from the outer mitochondrial membrane. This finding reveals a strategy used by HP-PRRSV to antagonize IFN-I antiviral responses. Our previous study has revealed that nsp4 mediates the cleavage of NEMO, and then blocks the NF-κB signaling pathway^30^. Considering that VISA locates upstream of IRF3 and NF-κB, targeting VISA might be more efficient for HP-PRRSV to suppress IFN induction and downstream signaling. Recently, Dong *et al*. reported that nsp4 of HP-PRRSV strain WUH3 cleaved human VISA at the residue Glu-268^52^. However, amino acid analysis shows that the homology between human VISA and porcine VISA is only about 55.4%, and the cleavage site on the human protein does not exist in porcine VISA. Since pigs are the only nature host of PRRSV, we investigated the effect of PRRSV on porcine VISA, which might reflect PRRSV-host interactions *in vivo*.

In comparison with the typical PRRS, the atypical PRRS caused by HP-PRRSV was characterized by quickly widespread, high fever, high morbidity, and high mortality in pigs of all ages^53^. The rapid molecular evolution of PRRSV results in a diverse composition of isolates with multifarious pathogenicity^54^. A series of studies have shown that different strains exhibit various effects on modulation of IFN-I antiviral responses. Most PRRSV strains have been shown to antagonize the induction of IFN-I^55^,^56^. However, a novel PRRSV isolate (A2MC2) induces the production of IFN-I and appears to have no inhibitory effect on antiviral response induced by IFNα^57^. Wang *et al*. reported that nsp1β of PRRSV strains VR-2332 and VR-2385 blocked ISGF3 nuclear translocation by inducing KPNA1 degradation, whereas Ingelvac PRRS modified live virus (MLV) had no effect on KPNA1^46^. Recent study revealed that HP-PRRSV nsp4 could display stronger inhibitory effect on IFNβ transcription than nsp4 of typical PRRSV isolates^36^. However, the precise molecule basis for this difference remains indistinct. In this work, we demonstrated that HP-PRRSV infection impaired VISA expression, while typical PRRSV strain CH-1a did not. In consistent with this observation, over-expression nsp4 of CH-1a exhibited no effect on VISA cleavage (Fig. 7D–F). These results indicated that the pathogenicity of PRRSV is likely related to its ability to evade host innate immunity. By comparing the amino acid sequences of HP-PRRSV strain JXwn06 nsp4 and the typical PRRSV strain CH-1a nsp4, we found there were 7 different amino acids, which might be responsible for the distinct effect on VISA. In addition, over-expression of CH-1a nsp4 in 3D4/21 cells suppressed IFNβ induction by blocking both NF-κB and IRF3 signaling pathways (Fig. 7A–C), suggesting that other strategies exist for nsp4 to inhibit IFNβ expression.

In conclusion, we identified that HP-PRRSV infection could antagonize IFNβ induction and signaling by reducing the protein level of VISA in infected cells. In addition, our data showed that nsp4 protein interfered with RLR antiviral signaling and IFNβ transcriptional activation by mediating VISA cleavage. Interestingly, we found that typical PRRSV strain CH-1a and its protein nsp4 had no effect on VISA. These findings might help us understand the molecular mechanisms of PRRSV immune evasion and develop countermeasures to control HP-PRRSV infection in the future.

## Materials and Methods

### Cells and viruses

HeLa and Marc-145 cells were cultured in Dulbecco’s Modified Eagle Medium (DMEM) (Gibco, Grand Island, NY), supplemented with 10% heat-inactivated fetal bovine serum (FBS) (Gibco, Australia) and 100 U/ml penicillin-streptomycin. 3D4/21 cells (ATCC number: CRL-2843), a porcine alveolar macrophage cell line, were maintained in RPMI Medium 1640 (Gibco, Grand Island, NY), supplemented with 10% FBS and 100 U/ml penicillin-streptomycin. Porcine alveolar macrophages (PAMs) were obtained from postmortem lung lavage of 8 week-old specific-pathogen-free (SPF) pigs, and maintained in RPMI Medium 1640 with 10% heat-inactivated FBS and 100 U/ml penicillin-streptomycin. All the cells were cultured and maintained at 37°C with 5% CO_2_.

HP-PRRSV strain JXwn06 (a highly pathogenic PRRSV strain isolated in Jiangxi Province; GenBank accession, EF641008.1) was propagated in PAMs, and typical PRRSV strain CH-1a (the first type 2 PRRSV strain isolated in China; GenBank accession, AY032626.1) was propagated in Marc-145 cells. Virus preparations were titrated on PAMs and Marc-145 cells, and then stored at −80°C until use.

### Plasmid construction

Porcine IFNβ-luciferase reporter plasmid was constructed using pGL3-Basic vector as described elsewhere^30^. Transcription factor IRF3, NF-κB and ISRE responding elements were synthesized and annealed to form double-stand DNA, and then separately cloned into pGL3-basic vector^58^. pRL-TK containing the Renilla luciferase was used as a normalization control. The protein expression plasmids pCMV-JXwn06-nsp4, pCMV-JXwn06-nsp4 mutants (3A, N69, MID, C157), pRK-Flag-RIG-I and pRK-Flag-IRF3 have been described previously^34^,^59^,^60^. To construct MDA5 and TBK1, cDNA fragments were amplified and cloned into pRK5-Flag at BamHI and SalI sites. To construct plasmid CH-1a-nsp4, fragment of PRRSV strain CH-1a cDNA was cloned into the pCMV-Myc vector at EcoRI and XhoI sites. cDNA encoding porcine VISA was amplified using reverse transcription PCR (RT-PCR) from total RNAs extracted from PAMs and cloned into pRK5-Flag at XbaI and HindIII sites. All the primers are listed in Table 1.

### Antibodies and reagents

Rabbit antibodies directed against TBK1 (D1B4), IRF3 (D83B9) and pIRF3 (4D4G) were purchased from Cell Signaling Technology. Rabbit anti-VISA (also known as MAVS, polyclonal antibody raised against residues 1–13 of MAVS), mouse anti-mitochondria (MTC02, recognizes a 60 kD non-glycosylated protein component of mitochondria) and mouse anti-c-Myc antibodies were obtained from Abcam. Rabbit polyclonal antibody to Flag-tag and mouse monoclonal antibody to β-actin were purchased from Sigma. Antibodies against Histone3 and α-tubulin have been previously described^61^. The antisera of nsp4 and GP5 were prepared by our lab. HRP-conjugated goat anti-mouse or anti-rabbit secondary antibodies for Western blot were purchased from Santa Cruz. FITC-conjugated goat anti-rabbit and TRITC-conjugated goat anti-mouse secondary antibodies for confocal microscopy were purchased from Jackson ImmunoResearch. Poly(I:C) was purchased from InvivoGen.

### Luciferase reporter assays

Marc-145 cells seeded in a 24-well plate were transfected with pGL3-IFNβ-luc, pGL3-IRFs-luc, pGL3-NF-κB-luc, pGL3-ISRE-luc, and pRL-TK using Lipofectamine LTX and Plus Reagent (Invitrogen). pRL-TK plasmid was used as a control for transfection efficiency. Six hours later, cells were mock infected or infected with HP-PRRSV at an MOI of 0.1. At 24 h after infection, cells were transfected with or without poly(I:C) for 8 h, and then harvested to determine luciferase activities. 3D4/21 and HeLa cells were transfected with a control plasmid or plasmids expressing RIG-I, MDA5, VISA, IRF3, and nsp4 along with pGL3-IFNβ-luc, pGL3-IRFs-luc, pGL3-NF-κB-luc, pGL3-ISRE-luc, and pRL-TK using Lipofectamine LTX and Plus Reagent (Invitrogen). At 24 h after transfection, cells were harvested for luciferase activity analysis.

Luciferase assays were performed with cell lysates prepared using the Dual-Luciferase reporter assay kit following the manufacturer’s instructions (Promega).

### RNA isolation and quantitative real-time PCR (qPCR)

RNA isolation and qPCR were performed as described previously^62^. Briefly, PAMs were infected with PRRSV at an MOI of 0.1 for 24 h, and then treated with or without poly(I:C) (10 μg/ml) for 6 h. Total RNAs were extracted with TRIzol (Invitrogen) and used for cDNA synthesis using M-MLV reverse transcriptase according to the manufacturer’s instructions (Takara). Quantitative RT-PCR (qPCR) analysis was performed using FastSYBR Mixture with ROX (Cwbiotech) on the ViiA^TM^7 real-time PCR System (Applied Biosystems). Gene-specific primers for Mx1, OAS, VISA and GAPDH were designed and listed in Table 1. The expression of Mx1 and OAS was normalized to glyceraldehyde-3-phosphate dehydrogenase (GAPDH) and presented as fold induction relative to the control. VISA expression was normalized to the mock infection.

### Western blot analysis

Whole-cell extracts were lysed in RIPA lysis buffer (Cwbiotech) supplemented with 100 U proteinase cocktail (Cwbiotech) and 20 μM phosphatase inhibitor. Cytosol and nuclear protein samples were prepared with Nuclear and Cytosol Fractionation Kit (Beyotime). Protein levels in each sample were quantified with BCA assay kit (Pierce Biotechnology, Inc.). Similar amounts of proteins from each extract were fractionated by SDS-PAGE and transferred to polyvinylidene difluoride (PVDF) membranes (Millipore). After blocking with 5% milk in PBS with 0.05% Tween-20 (PBST), membranes were incubated for 2 h at room temperature with the primary antibodies at a suitable dilution as recommended (anti-TBK1, IRF3, p-IRF3, VISA and Histone3 at 1:1000; anti-β-actin, α-tubulin, Flag and c-Myc at 1:2000). The membranes were then incubated with the appropriate secondary antibodies for 1 h at a dilution of 1:10,000. The antibodies were visualized by use of the ECL reagent according to the manufacturer’s protocols.

### Confocal microscopy

HeLa cells cultured in microscope cover glass (Fisher Scientific) were washed with ice-cold phosphate buffered saline (PBS) and fixed with 4% paraformaldehyde. Cells were permeabilized with 0.2% Triton X-100 for 3 min and blocked with 1% bovine serum albumin (BSA) for 1 h at room temperature (RT). Cells were then incubated with the indicated primary antibodies (anti- mitochondria and Flag at 1:200 dilution) for 1 h at RT. Following washing, cells were incubated with proper secondary antibodies (FITC-conjugated goat anti-rabbit or TRITC-conjugated goat anti-mouse IgG) for 45 min at RT, washed and stained with 4, 6-diamidino-2-phenylindole (DAPI) to detect nuclei. Immunofluorescence was performed with a Nikon A1 confocal microscope.

### Statistical analysis

Results are presented as means ± SD for at least three independent experiments. Data were analyzed by GraphPad Prism software using Student’s *t* test. Differences in data were considered to be statistically significant if the *P* value is less than 0.05. *P < 0.05; **P < 0.01; ***P < 0.001.

## Additional Information

**How to cite this article**: Huang, C. *et al*. Highly Pathogenic Porcine Reproductive and Respiratory Syndrome Virus Nsp4 Cleaves VISA to Impair Antiviral Responses Mediated by RIG-I-like Receptors. *Sci. Rep.* **6**, 28497; doi: 10.1038/srep28497 (2016).

## Figures and Tables

**Table 1:** Primers and sequences used in qPCR and cloning.

Primers	Sequence (5′-3′)
Mx1-F	CACAGAACTGCCAAGTCCAA
Mx1-R	GCAGTACACGATCTGCTCCA
OAS-F	AGCAAGGAAGCAGGAAAACA
OAS-R	GCTTCCCAGAAGATGCAAAG
VISA-F	AAAGTGCCTACTGGCTTGCT
VISA-R	TGCTGGAGTCTCCTTTTCAGG
GAPDH-F	CCTTCCGTGTCCCTACTGCCAAC
GAPDH-R	GACGCCTGCTTCACCACCTTCT
TBK1-F	CCG GGATCC ATGCAGAGCACTTCTAATC
TBK1-R	CGC GTCGAC AAGCTAAAGACAGTCAACG
MDA5-F	CCG GGATCC AGAAAGATGTCGAATGGGTAT
MDA5-R	CGC GTCGAC TCTTCAATCAAGTGCTAATCC
sus-VISA-F	GC TCTAGA ATGACGTTTGCCGAGGACAAG
sus-VISA-R	CCC AAGCTT AGGACAATGGGACCGAAATG
CH-1a-nsp4-F	CG GAATTC GGGGCGCTTTCAGAACTCGAA
CH-1a-nsp4-R	CCG CTCGAG CATTTCCAGTTCGGGTTTGGCA
